# Mouse models for human intestinal microbiota research: a critical evaluation

**DOI:** 10.1007/s00018-017-2693-8

**Published:** 2017-11-09

**Authors:** Floor Hugenholtz, Willem M. de Vos

**Affiliations:** 1 Laboratory of Microbiology, Wageningen University, Stippeneng 4, Building 124, 6708 WE Wageningen, The Netherlands; 2 Research Programme Unit Immunobiology, Department of Bacteriology and Immunology, Helsinki University, P.O. Box 21, 00014 Helsinki, Finland; 3 Present Address: Division of Infectious Diseases, Department of Medicine, Academic Medical Centre, University of Amsterdam, Amsterdam, The Netherlands

**Keywords:** Microbiome, Metagenome, Phylogeny, Murine models, Reproducibility, Diet

## Abstract

Since the early days of the intestinal microbiota research, mouse models have been used frequently to study the interaction of microbes with their host. However, to translate the knowledge gained from mouse studies to a human situation, the major spatio-temporal similarities and differences between intestinal microbiota in mice and humans need to be considered. This is done here with specific attention for the comparative physiology of the intestinal tract, the effect of dietary patterns and differences in genetics. Detailed phylogenetic and metagenomic analysis showed that while many common genera are found in the human and murine intestine, these differ strongly in abundance and in total only 4% of the bacterial genes are found to share considerable identity. Moreover, a large variety of murine strains is available yet most of the microbiota research is performed in wild-type, inbred strains and their transgenic derivatives. It has become increasingly clear that the providers, rearing facilities and the genetic background of these mice have a significant impact on the microbial composition and this is illustrated with recent experimental data. This may affect the reproducibility of mouse microbiota studies and their conclusions. Hence, future studies should take these into account to truly show the effect of diet, genotype or environmental factors on the microbial composition.

**Electronic supplementary material:**

The online version of this article (doi:10.1007/s00018-017-2693-8) contains supplementary material, which is available to authorized users.

## Introduction

In adult life, a healthy human may harbor several hundreds of different microbial species in their intestine, which collectively encode more than 100-fold more non-redundant genes than there are in the human genome [1–3]. The composition of the intestinal microbiota is driven by factors such as diet, antibiotic therapy, maternal microbiota and genotype [4–9]. Since the intestinal tract is the main point of contact of the host immune system and microorganisms, the role of microbiota in both local and systemic immune function plays an important role in immunity and health [10]. Early comparative analyses of the intestinal microbiota of human and other animals have shown that each mammalian species harbors a distinct microbial composition and can be grouped based on their microbial community and diet [11]. Carnivores, omnivores and herbivores could be distinguished by increasing microbiota diversity, which probably reflects the large variety of plant-derived carbohydrates in the diet of herbivores. The differences in composition and diversity of intestinal tract microbiota in these animal groups indicate that both diet and host collectively affect the microbial composition [11–13].

Studies of the local microbiota at different locations along the human intestinal tract require rather invasive sampling methods. Pioneering studies have used these and provided the first molecular biogeography of the human intestinal microbiota by addressing colonic and ileal sites [14, 15]. However, these approaches cannot be scaled for practical and ethical reasons. While ethical considerations nowadays also apply to rodent models, these provide an easy way to collect many samples from different sites, allow multiple comparisons at large scale, and have the great advantage to offer a wide range of different genotypic backgrounds. Moreover, rodents and specifically mice have become the most studied disease models for pharmaceutical research [16]. Mice models have also been used to study the interaction of intestinal microbes and its host since the early days when large scale studies became feasible due the development of molecular and high throughput approaches [17]. Initially, most attention has focused on germ-free mice models and provided basic insights in initial host–microbe interaction [18]. However, increasingly mice are used as models to study dietary effects, disease development, and the impact of microbial therapies. However, in order to translate such generated knowledge from mouse to man, the similarities and differences between their intestinal microbiota need to be considered and these are reviewed here with specific attention to the historic development of inbred mouse models, the impact of genomics and the difference in intestinal anatomy.

## History of mouse models

**Fig. 1:**
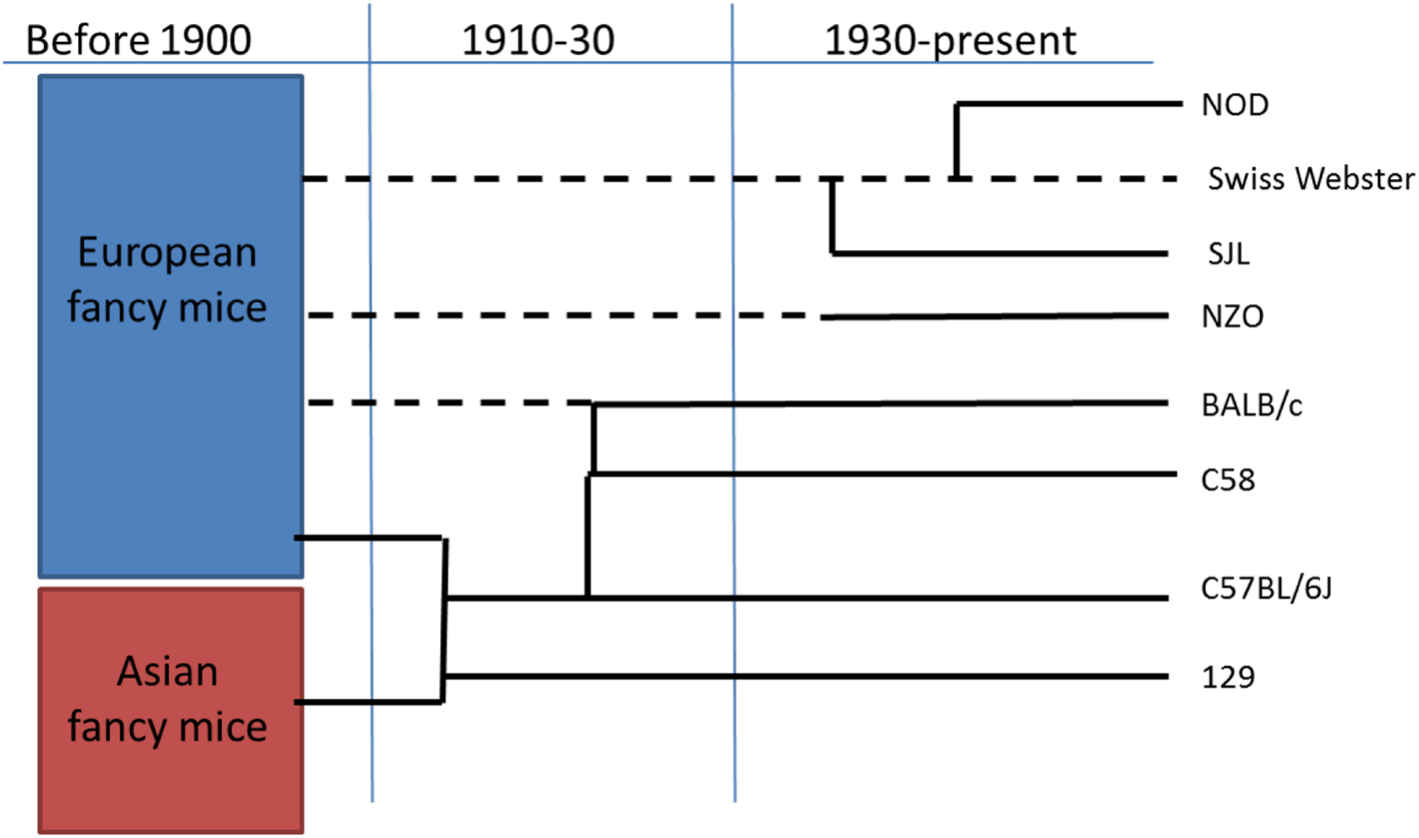
A simplified family tree of the main mouse strains used in intestinal microbiota research. Solid lines indicate inbreeding and dotted lines indicated outbreeding of a mouse line. When lines are connected a cross or a new line was created by selection. Data adapted from [20, 21]

The majority of presently employed murine strains, i.e., strains belonging to the species *Mus musculus,* have a common origin that goes back over 100 years ago and derive from Asian or European fancy mice, usually yellow, white or with another appealing color, that had been developed as pets as early as 1200 BC in China (Fig. 1). The best recorded example of an anecdotal development is that of Miss Abbie E.C. Lathrop who started breeding mice in the early 1900s in Granby MA, USA [19]. In a couple of years, her business had grown into an operation with over ten thousand mice that were used to be sold as pets but also provided laboratories in the area for scientific studies. Subsequently, she also started inbreeding mice, avoiding mixing her mice with wild mice [20, 21]. These inbred mice are the ancestors of the most commonly used strain C57BL/6 created in William Castle’s lab (Fig. 1). Since around 1910, these mice were inbred, with over two generations per year; thus, many of the presently available mice have been inbred for over 150 generations on average [22]. Also the 129, C3H and BALB/c have a similar origin, where the latter two were a cross between progenitors of the C57 line and Bagg albino’s from H. Bagg (Fig. 1). Some strains were developed much later, like the NOD inbred strain, which was derived from an outbred colony of Swiss Webster mice. Ohtori developed the inbred CTS strain from this colony [18]. And in 1980, the F6 of the CTS strain selected for diabetes was taken separate and the F20 developed spontaneous insulin-dependent diabetes, and named NOD [23]. The advantages of using mice models appealed to many scientists, from that time till now, since they are relatively small, easy to maintain in large numbers, and can be inbred or genetically modified to be used as models to study human diseases. It has been estimated that presently over 90% of the rodents used for pharmaceutical research are mice [16].

## The mouse versus the human genome

Lineages of men and mouse are separated by more than 90 million years of evolution, yet more than 85% of the genomic sequences between mouse and human are still conserved [24, 25]. One of the major divergences that occurred in the genomes in the course of this evolutionary time span is found in the primary sequence of regulatory elements. Recent detailed genomic and transcriptomic analysis revealed that half of the transcription factor binding sites of the murine genome does not appear to have orthologous sequences present in the human genome [26]. However, the regulatory networks among transcription factors are highly conserved between mice and humans [27]. In contrast, the overall gene expression and its regulation were found to be considerably different between the two species. However, it should be emphasized that most human functional studies have been performed with cell lines and it is known that their expression patterns may differ from the large variety of tissue-specific expression in the human body. Comparative genomic studies do not suffer from that bias and have shown that the immune system and its regulation has dramatically changed during evolution, indicating a rapid but species-specific adaption of this system in the different species [28]. As the intestinal tract is the site of the majority of innate and adaptive immune interactions, these large immune differences between mice and man may provide a perspective on failing extrapolations of many mouse studies on inflammatory and immune diseases [29].

## Comparative physiology of the intestinal tract in mouse and man

Nowadays, mice belonging to the species *M. musculus* are often used to systematically study the roles of the diet, pathogens and/or the influence of the host genotype on microbial diversity in GI tract and to relate this back to the human situation [22].

**Fig. 2:**
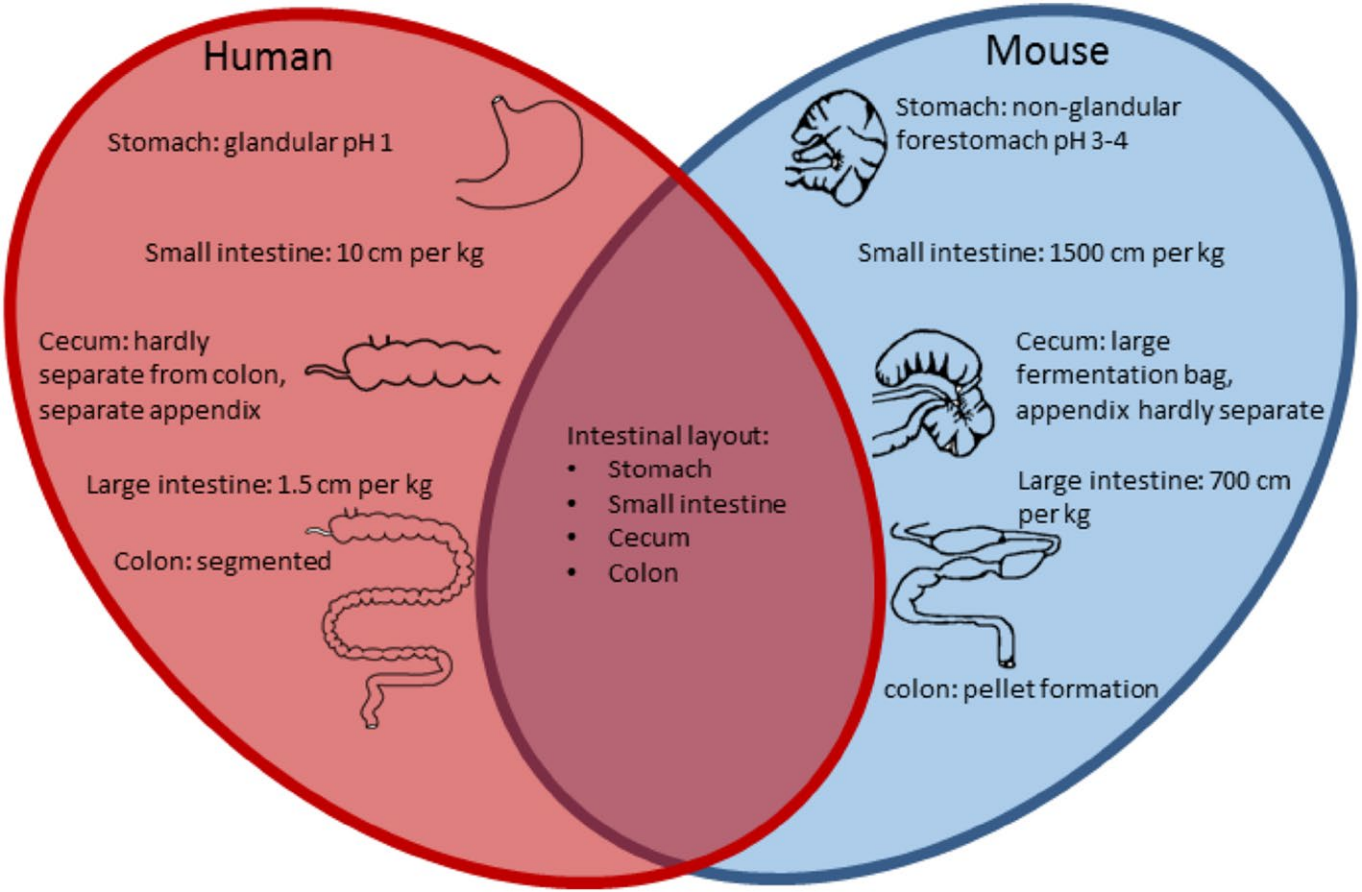
Comparison of the intestinal tract features of human and mouse. The main similarities and differences are listed in a Venn diagram [37–39]

Mice are exclusive herbivores, while humans can be herbivores, carnivores and everything in between, depending on culture, food supply and many other factors. It appears that there are considerable anatomical, histological and physiological features of the intestinal tract that are shared. The main difference is the size of the intestinal tract in relation to the total size of the species but there are many distinct differences throughout the intestinal tract, which should be considered during experimental design and interpretation (Fig. 2).

One of the most remarkable differences to be noted at the beginning of the tract, is the presence of a non-glandular forestomach in mice that is absent in humans. This forestomach is lined with keratinizing squamous mucosa and covers two-thirds of the entire stomach. The forestomach has no secretory activity and is used for food storage [30] and is covered with a biofilm comprised of strains of various *Lactobacillus* spp. [31, 32]. Although *Lactobacillus reuteri* and *Lactobacillus johnsonii* are found throughout the mouse intestinal tract, there is a strong indication that the forestomach is their main habitat and that the cecal populations are composed of cells that have descended from the forestomach populations [33]. Comparative genomic analysis has shown that the murine *L. reuteri* strains are very different from those found in humans and have urease genes to cope with low pH and a variety of rodent-specific genes which, when inactivated, affects their persistence in mice [34].

The remaining third of the mouse stomach is the glandular stomach, which is similar to that of man. However, there are major differences in the fate of food in the stomach. Gastric emptying in humans proceeds linearly, with a half time of 30 min (empting rate of 1.64% per min), whereas gastric emptying of the mouse has an exponential decay with a decay constant of 77 ± 17 min and a half time of 34 min. Differences in eating behavior, feeding patterns and biorhythms between mouse and human can explain this difference since mice forage and feed almost continuously at night while humans consume most of their foods during daytime when their stomach is empty. Hence, in the mouse stomach, freshly eaten food particles are constantly mixed with gastric fluids diluting the present bolus [35]. This might be the reason why the range of pH is smaller in the mouse stomach, from pH 2.7–4.1, while in humans it can go down to pH 1. This relatively high pH in the mouse stomach probably also enables the formation of biofilms of *Lactobacillus* spp., while in humans the stomach is colonized by mainly Streptococci, *Prevotella* spp. and *Helicobacter pylori,* that are likely to be acid adapted [36].

The small intestine is the longest part of the GI tract, approximately 33 cm in mice and 700 cm in humans and is divided in three regions. There are various ways to compare the impact of these size differences and while these can be related to length, body surface or blood volume, in most cases the simplest comparison is that with the weight. A mouse weighs around 0.02 kg while the weight of an average human being is ~70 kg; so the length of the small intestine per kg is in mice 1500 cm per kg and humans 10 cm per kg (Fig. 2). The duodenum is the most proximal region, where bile and secretory products of the pancreas enter the intestinal lumen. The next part of the small intestinal tract is the jejunum followed by the ileum. The outer mucosa layer of the small intestine differs the most between human and mouse. The overall appearance of the mouse mucosal surface is smooth, while the human mucosal contains circular folds, known as *plicae circularis*, to increase the surface area [37]. This specific anatomy of the human small intestine provides a niche for mucus-associated bacteria, which is not present in mice and hence could, therefore, be an important difference, influencing microbial composition. Similarly, the architecture of the villi varies through the small intestine with distinct differences in mice and man. In both the duodenal villi have a leaf-like structure but in mice these change to a more cylindrical shape in the jejunum and ileum. In contrast, in the human jejunum the villi become taller with a more frond-like structure and they become thinner and sparser in the ileum.

The large intestine is up to 14 cm long in mice and 105 cm in humans and can be divided into the cecum and colon. The cecum of mice is relatively large, being 3–4 cm in length, and functions as a microbial fermentation vessel, while in humans it is 6 cm and of minor importance. Expressed per kg of body weight the length of the large intestine is in mice 700 cm per kg and humans 1.5 cm per kg, while the cecum is in mice 175 cm per kg and in humans 0.086 cm per kg (Fig. 2). This illustrates that, relative to body weight, the large intestine is a much larger organ in mice than in man. Both humans and mice have a cecal appendix, although it is not a pronounced separate section in mice as it is in humans [38]. Moreover, the human colon is segmented, with pouches called haustra, while the mouse colon has a smooth serosal appearance. The proximal colon of the mouse has a mucosa with transverse folds. Halfway, the colonic mucosa is flat and in the distal colonic mucosa there are longitudinal folds, while the human colonic mucosa has transverse folds throughout the colon [39].

The overall intestinal transit time is known to affect the intestinal microbiota. After human consumption of a meal, the transit takes 14–76 h, a wide range due to dietary and population differences. The type of diet has a major impact on the transit time, and resistant starch increases transit time by almost 20 h compared to fully digestible starch [40]. In mice the total transit time is only between 6 and 7 h, up to ten times as fast as humans. This is compatible with the total metabolic rate that is approximately seven times higher in mice as compared to man when corrected for body weight (see below).

The mucus layer is important for the protection of the intestinal tract. It forms a physical network, providing a barrier between bacteria and host, minimizing contact between bacteria and epithelial cells [41]. Defects in the mucus layer have been linked to various human diseases, such as inflammatory bowel disease (IBD), and the mucosa of IBD patients harbors a higher number and different species of bacteria than that of healthy subjects [42–44]. Notably in this respect is the increase of potential pathogenic *Ruminococcus torques* and *Ruminococcus gnavus* at the cost of *Akkermansia muciniphila* in IBD mucosa [45]. It has been shown that mucus layer thickness is compromised in IBD and that this may impact on its functional organization. A recent comparative study addressed mucus thickness, penetrability, and proliferation rate using live tissue explants of human and mouse colon in a perfusion chamber [46]. The mucus growth rate was shown to be higher in the human colon (240 ± 60 µm per h) as compared to that in the murine colon (100 ± 60 µm per h). Furthermore, the final mucus layer was demonstrated to be thicker in the human (480 ± 70 µm) as compared to the mouse colon (190 ± 40 µm). Mucus penetrability was similar in mice and humans, since fluorescent beads with a diameter of 1 µm in both species penetrated the outer 40% of the colonic mucus layer, while the inner 60% was impenetrable for the beads [46]. However, it is possible that specific intestinal microbes adapted to the mucus behave differently than on these model beads and it has been found that *A. muciniphila,* a well-established mucus utilizer, may have a size as small as approximately 0.5 µm depending on the growth medium [47].

The major structural Muc proteins in the inner and outer mucus layers are the same in mouse and man, represented by Muc2 in the small and large intestine and Muc5AC in the stomach. The outer mucus layer is looser than the inner layer, because of proteolytic cleavages due to host proteases and microbiota in mice and men [41]. However, there are some noticeable differences between mice and humans in mucin composition at the molecular level. Specifically, the monomeric Muc2 protein has a different size in human and mice (5179 versus 2680 residues, respectively). It possesses a large and a small domain, both of which are rich in proline, threonine and serine (and are therefore called PTS domains), but while the human large PTS domain consists of an almost perfectly tandem repeat of 23 amino acids, that of the mouse is not repetitive [48]. Disulphide bonds amplify the size of the mucin monomers while O-glycosylation results in O-glycan molecules extending in all directions of the PTS domain, making the molecules look like a bottle brush and giving mucin its gel-forming properties through high capacity of binding water. Obviously, this post-translational glycosylation and hence biophysical properties differ between the Muc2 molecules from mice and man but their details have not been addressed as there are hundreds of different glycan structures. What is known is that the primary glycosyltransferases involved in the extending and branching of the O-glycan molecules differ and involve the core 1 β1,3-galactosyltransferase (C1galt1) in mice and the core 3 β1,3-*N*-acetylglucosaminyltransferase (C3GnT) in humans [49]. Apart from these core enzymes that differ between mouse and man, it is likely that other glycosyltransferases may also vary as do the sialidation and sulfonization processes that are particularly prominent in the colon where they protect the mucus from rapid microbial degradation. These modifications mask the glycan profile which is reflected by the blood group status, which is evident in the stomach and small intestine. The reason for this could be that the glycan composition is important for the selection of commensal microbiota [50]. Fucosylation, however, is a glycan modification which is known to occur in mice and man in a similar manner [51]. In humans fucosylation is determined by the FUT2 gene, the expression of which is affected by the gut microbiota, especially during colonization [52]. Genetic polymorphisms that affect fucosylation have an impact on the microbiota in human, particular on the bifidobacterial composition as well as on the abundance of *Bifidobacterium, Bacteroides* and *Akkermansia* spp., all potentially mucus-degrading bacteria [53, 54]. Bacteria often carry adhesins that can bind mucins, which serve as an adhesion substrate and nutrient source [49]. This could be an explanation of the difference of mucus-associated bacteria between humans and mice. However, *A. muciniphila*, a specialized mucus-utilizing bacterium is almost identical in mice and humans [55], indicating that even though there are differences in the mucus, this species, and probably others, do not need to be very different to proliferate on varying mucus compositions.

Overall mice show lower intestinal pH values, oxygen tension levels and a different glycan profile in the mucus than humans, aspects that are likely to be, at least partially, responsible on the observed differences in microbial composition [56–58] (see also below).

## Energy saving strategies

Small animals, with a high metabolic turnover rate, need to digest more food per body mass than larger animals and it has been calculated that an average adult mouse has an approximately sevenfold higher metabolic turnover rate as compared to the average adult human [59]. Mice eat, therefore, around the clock, but mostly during the night, which is their active time, exposing intestinal tissue to different microbes and metabolites as the day goes by and hence affecting the circadian rhythm of the host [60]. With obviously different synchronicity this may also occur in human where links between circadian rhythm and intestinal microbes have been suggested in a longitudinal study [61]. Because of their higher energy demands, small animals need to have a short retention time of foods, especially when the digestibility of the food is low. The generation interval of gut microbiota (human or murine) needs to be 0.69 times the retention time to maintain a population of the same numerical size and to prevent washout [59]. Some rodent species depend on separation mechanisms to maintain microbiota in their cecum, but allow food particles to pass on quickly [62]. In mice, a slight delay of flow of fluid digesta is observed compared to particle digesta. A separation mechanism depending on mucus, called “the mucus trap”, is present in the mouse. The mucus trap is folds in the proximate colon, that creates a furrow, where a mixture of bacteria and mucus can be transported back to the cecum [63]. So it appears that mice partly recycle their microbiota as a sort of colonic transplantation. An ultimate form of this recycling is found in coprophagy, the behavior by which feces is re-ingested. This is practiced by mice and contributes to the nutritional value of their diet by ensuring that vitamin K, some B vitamins, and short chain and other fatty acids that are produced by microbiota in the cecum, are not lost by defecation, but re-enter the murine intestine to be absorbed [63]. Coprophagy is known to affect the intestinal microbiota within litters and can be avoided by cages equipped with grids, but coprophagy is still considered as an important difference between human and mice [64].

## Murine versus human microbiota

The phylogenetic makeup of the bacterial communities in both human and mouse seems to be similar at phylum level, where the two main bacterial phyla of the murine intestinal tract are the *Bacteroidetes* and the *Firmicutes* [65, 66]. However, this also applies to many other mammals, herbivores and carnivores alike [65, 66]. Several obvious differences between the intestinal tract of mouse and man received considerable attention. The murine intestinal tract was found to harbor large amounts of members of the phylum *Deferribacteres*, which in human are only found in minute amounts in the stomach [36], and the main species of this phylum is *Mucispirillum schaedleri* [67], which colonizes the mucus layer in mice. Moreover, mice harbor a specific member of the *Firmicutes* with an unusual morphology, the segmented filamentous bacteria (SFB), also termed ‘*Candidatus arthromitus’* [68], which have a pronounced effect on the maturation of the innate immune system [69–71]. SFB have been thought to be lacking in humans but a recent very deep analysis provided support for their presence in some human infants during the first 3 years of life, although no functional studies have yet been performed that would support a similar role in immune maturation as for their murine counterparts [72].

A recent comparative survey of the phylogenetic composition of 16 human subjects and 3 often used mouse lines indicated that their microbiota looks alike but is quantitatively very different [73]. Around 80 microbial gut genera were reportedly shared between mouse and man, and this number was recently confirmed in a comparison of murine and human 16S rDNA datasets [74]. However, there are considerable variations in the genera that were observed in the mouse data sets and for instance *Faecalibacterium, Succinivibrio* and *Dialister* were not found in some laboratory mice [73, 75], while they were detected in other more comprehensive study [74]. A trivial but important explanation for this is the use of different mouse strains and providers (see below), but other reasons for the observed discrepancies are differences in analysis and specifically its depth since different approaches were used to address the microbial composition, including different 16S rRNA gene-based primers, targeted variable regions and sequencing platforms [74]. Hence there is a need to assess these and other differences between the human and mouse microbiota with large datasets that are generated using exactly the same protocols.

**Fig. 3:**
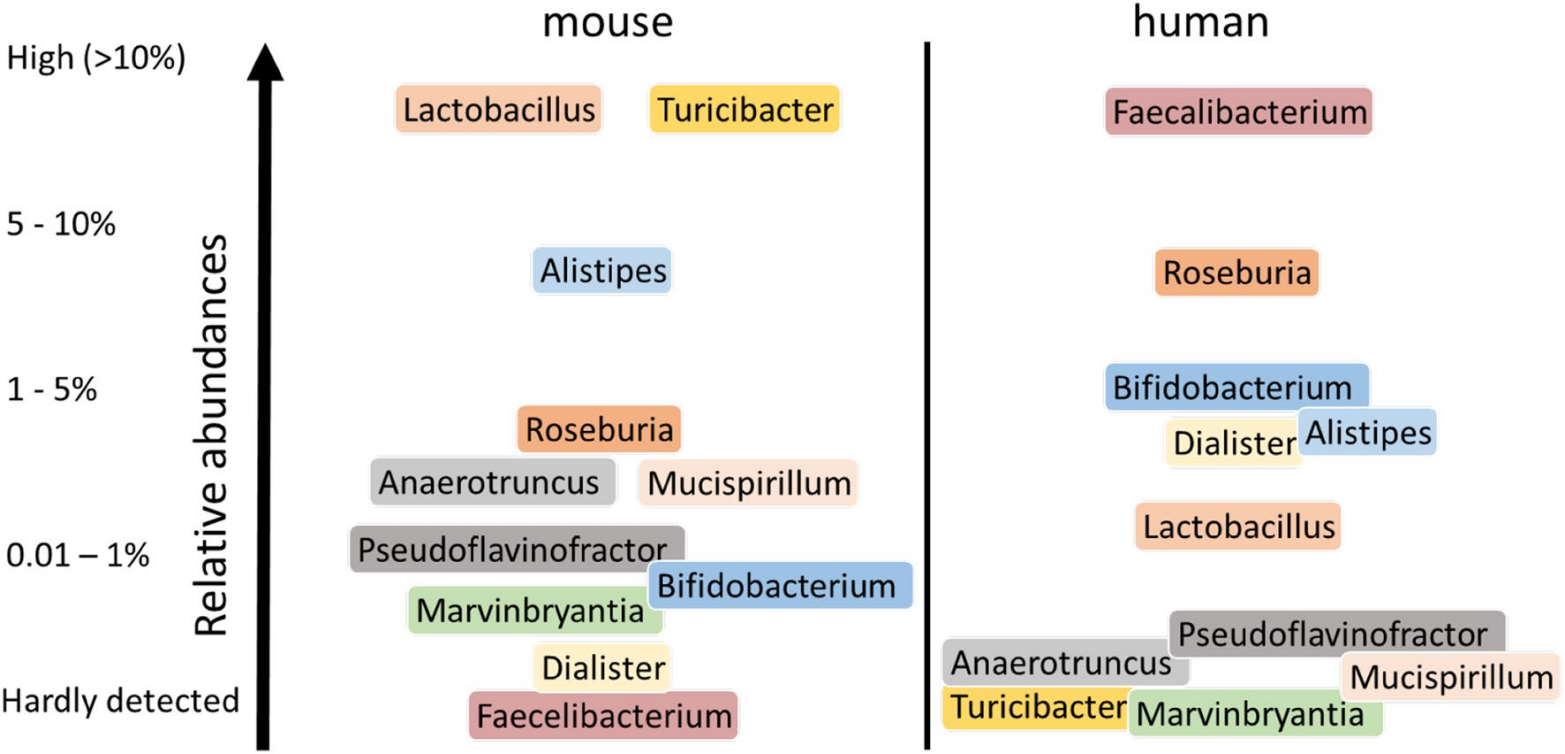
Major different human and murine intestinal genera. Only genera are shown that showed consistent differences in relative abundance between humans and mice [73, 74, 76]

Recently, an extensive mouse microbiome catalog was made available through deep metagenome sequencing, which obviates some issues associated with phylogenetic approaches [76]. Moreover, these mouse metagenomic datasets can be easily compared with the human metagenome baseline that has been collected in recent years [76]. This comparison confirmed that the human and mouse intestinal microbiota show considerable similarity at the genus level but reveal large quantitative differences (Fig. 3). Moreover, a total of 60 genera were detected in the mouse gut microbiome core, of which 25 were shared with the core genera in the human gut microbiome, where the core was here defined as genera being present in all samples. When the mouse microbial genes were compared with that found in human, only 4% were found to share 95% identity and a coverage of 90%. Remarkably, almost 80% of the annotated functions were common between the two datasets, indicating significant functional overlap. However, while over 1500 species have been isolated from the human gut, from which over half have been deposited [77, 78], only around 100 species have been cultured from mice strains and deposited, most only very recently [79]. Hence, the majority of mouse gut bacteria remain to be cultured and characterized. It should be noted, however, that strain analysis is the next level that needs to be addressed as mouse and human strains of the same species may differ considerably, as is exemplified by the strains of *L. reuteri* that appear very different between mice and man as indicated above.

## Currently used mouse strains

A large number of different strains of mice are available, especially when considering the number of genetically modified mice. Over 400 inbred strains have been described and their genealogies categorized (reviewed in [22]). Most of the widely used model strains can be traced back to the last century (Fig. 1). The advantage of the inbred strains is their genetic similarity that contributes to the reproducibility of the experimental approaches. Most inbred strains originate from either the *Mus musculus domesticus* or *M. musculus musculus* and show considerable genetic and phenotypic similarity [20, 80]. However, the inbred strains are very different from wild-derived mice and the microbiota from the wild wood mice *Apodemus sylvaticus* has been shown to be subject to strong seasonal shifts in gut microbial community structure, potentially related to the transition from an insect- to a seed-based diet [81]. Such fluctuating environmental factors do not affect captive mice that receive a similar diet over time.

## Microbiota in mouse strains—impact of diet

In most studies with disease models, germ-free systems or dietary interventions, use is made of inbred strains. Some have specific properties, such as the C57BL/6 mice that develop an obese phenotype, together with obesity-related diseases, after several weeks of a high-fat diet. Hence C57BL/6 mice are often used in studies related to diet-induced obesity, type 2 diabetes and atherosclerosis [82, 83]. It was the C57BL/6 mice that were used in the pioneering study where the intestinal microbiota of obese mice together with the corresponding phenotype could be transferred to germ-free C57BL/6 mice, providing the first evidence for a causal contribution of the intestinal microbiota on obesity [84]. Humanizing these mice with human microbiota seemed quite successful: 88% of the genus-level taxa were found in the mice and in the donor samples [85]. Humanized mice obtained using this technique have been applied not only to study obesity but also for instance metabolic disorders, alcoholic liver disease and infectious diseases [85–87]. To the best of our knowledge, this experimental approach has not been reproduced in other mouse strains and considering the large variety in mouse strains and their microbiota, it should be kept in mind that extrapolation to the human system is a considerably larger step than reproducing this in other mouse lines. A highly relevant study revealed that the intestines of BALB/c and NIH Swiss mice, which differ markedly in behavior, show different microbial composition, which could be transferred by microbiota transplantation to germ-free derivatives. Remarkably, these mice adopted not only the microbiota but also the behavior from the donor strain as was evident from stress tests [88].

An important confounder has shown to be the housing of mice. In some cases, complete phenotypes disappeared after a mouse house was renovated or renewed. In some cases, this could be tracked down to the microbiota that had changed and apparently was involved in the phenotype as reported recently [89]. The housing effect seems even to be larger than the effect of the genetic background [76, 90, 91]. However, what the effect the birth mother has on microbiota composition is at the moment under debate since in some studies the genotype (mouse strain) of the mouse had a more pronounced effect on the microbiota development than the genotype of the birth mother [90, 92].

So far only a few studies have shown difference in microbial abundances between different mouse strains. Two independent studies showed the abundance of the genera *Akkermansia, Alistipes* and *Lactobacillus* to be significantly different in C57BL/6, BALB/c and NOD mice, although to a different extent [74, 76]. However, the number of studies comparing the microbiota between mouse strains is still limited and would benefit from more comparative studies. Efforts to diminish the genotype effect on gut microbiota in mice by intercrossing inbred strains resulted in high inter-individual variation of the microbiome after 4 generations but the inter-individual variation became less after ten generations [93].

**Fig. 4:**
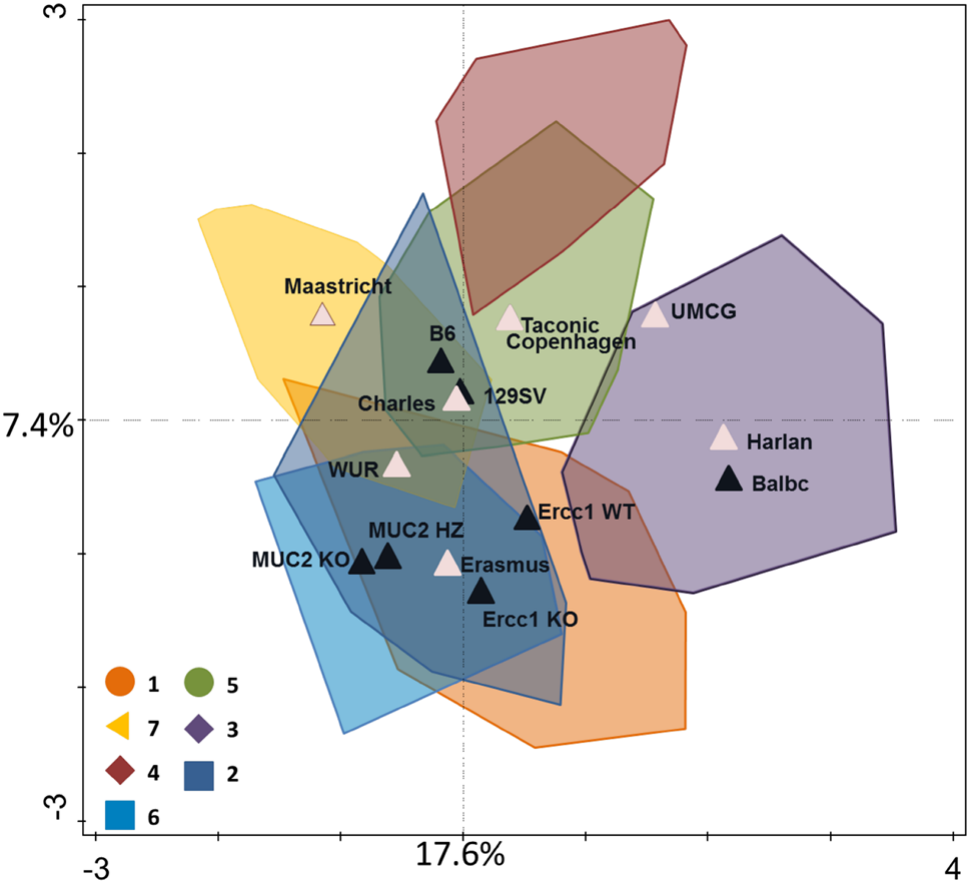
Redundancy analysis of the large intestine samples of seven studies, containing a total of 244 samples [44, 94–100]. Genotype, facility and provider are taken along as variables for the analysis and explain 43.5% of the data. Colors 1–7 are per cohort, black triangles indicate the centers of the different mouse genotype variables and pink triangles indicate the centers of providers and facilities variables. Here the level of clustering per cohort is less than on probe level (Supplementary Fig. S1) and the facility Wageningen University and different providers (Supplementary Fig. S2A) explain a significant proportion of the data over the effect of the strain C57BL/6J (Supplementary Fig. S2B), which comes fourth in percentage that it can explain as a variable in the data. In Table 1 are the significant variables shown

There is not a specifically recommended mouse model for dietary interventions and often use is made of strains for which there is in-house experience or that are easily commercially sourced. This may not be a desirable situation since recent studies have shown that environmental factors and the genetic background of mice have a significant impact on the microbial composition [75]. To illustrate the effect of genotype, cohort, provider and housing facilities on the gut microbiota of mice we have carefully analyzed eight different mouse studies with dietary interventions [44, 94–100]. The microbiota was analyzed using an identical microbiota analysis pipeline based on a phylogenetic microarray developed and benchmarked previously [101]. This closed system enabled us to compare multiple studies over time in exactly the same way and provide a read out at the species level. The mice in this analysis came from nine different studies (cohorts) where feces was collected and included three inbred strains—C57BL/6J, BALB/c and 129Sv—both genders, young and old mice and were housed in four different facilities. This analysis showed a larger effect of the cohort than the genotype of the mice, the provider, gender or the housing facility (Supplementary Fig. S1). To address whether the same effect occurs at genus level, the same samples were analyzed in redundancy analysis [102]. This revealed a clear effect of the facility and provider (Fig. 4; details in Supplementary Fig. S2A and S2B). In conclusion, this study provides an unbiased indication that cohort and facility have a larger effect on the microbiota than the mouse genotype, confirming recent murine metagenome analyses by Xiao et al. [76]. It also indicates that all used mouse strains are good candidates for dietary interventions.

**Table 1:** Significant variables of the redundancy analysis

Variable name:	Percentage of variation explained	*P* value
Facility WUR	13.1	0.002
Provider Harlan	7	0.002
Provider Maastricht	6.9	0.002
Strain B6	4.5	0.002
Provider Charles River	3.5	0.002
Facility UMCG	2.7	0.002
Strain Ercc1KO	2.2	0.002
Strain Ercc1WT	1.4	0.002
Strain BalbC	1.3	0.002
Strain 129SV	0.7	0.01

*P* values are calculated by Monte Carlo permutation, variables are ordered by importance of percentages of variation they can explain

## Conclusions

Mice are often used to systematically study the impact of the diet and other environmental factors as well as the host genotype on microbial diversity in intestinal tract and to relate this back to the human situation. While mice and humans have many similar anatomical, histological and physiological features in their intestine, there are very large differences in size, metabolic rate and dietary habits. Hence, it is no surprise that there are large differences in the intestinal microbiota not only in the qualitative representation of taxa but notably in their quantitative contribution. Altogether, only a few percent of the bacterial genes are shared between mice and man, and a notable example is the presence of the biofilm of *Lactobacillus* spp. in the forestomach of mice. In view of these results, one may wonder why mouse models are used so often for translation to human and the simple answer could be that there is no better alternative.

It also has been shown that there are considerable differences in microbial composition between mouse strains. Hence, it is striking to note that many dietary interventions or pioneering studies have not been reproduced in other mouse strains. Our analysis and other recent studies clearly indicate that the provider and housing conditions are also important factors to take into account, especially when results of other studies are compared [76, 103]. Hence, extreme care should be taken when comparing results of mouse studies from mice of different providers and handled in different facilities. Future studies should focus on reproducing microbial differences at different locations with different mouse strains to truly show a robust effect of the diet, genotype or environmental factors on the microbial composition. Since the human intestinal microbiota is so different from that of mice, such robustness checks should precede any extrapolation to human.

### Electronic supplementary material

Below is the link to the electronic supplementary material.

Supplementary Figure S1 Clustering of samples by the probe level of the MITChip. This DNA oligonucleotide microarrays target the V1 and V6 variable regions within the 16S rRNA gene sequences of the intestinal microbiota, allowing the comprehensive profiling of intestinal microbiota composition [82, 101, 104–106] (PDF 67 kb)

Supplementary Figure S2. Redundancy analysis of the large intestine samples of seven studies, containing a total of 244 samples. Genotype, facility and provider are taken along as variables for the analysis and explain 43.5% of the data. Here the clustering is shown of the different providers (A) and of the strains (B). In Table 1 are the significant variables shown (PDF 127 kb)
