# Supplementary material for: Mouse models for human intestinal microbiota research: a critical evaluation
Source: Cell Mol Life Sci. 2017 Nov 9;75(1):149–60. doi: 10.1007/s00018-017-2693-8 (PMC5752736; doi:10.1007/s00018-017-2693-8)
Supplement: Supplementary file 1 — Supplementary Figure S1 Clustering of samples by the probe level of the MITChip. This DNA oligonucleotide microarrays target the V1 and V6 variable regions within the 16S rRNA gene sequences of the intestinal microbiota, allowing the comprehensive profiling of intestinal microbiota composition [82, 101, 104–106] (PDF 67 kb) [file 18_2017_2693_MOESM1_ESM.pdf]

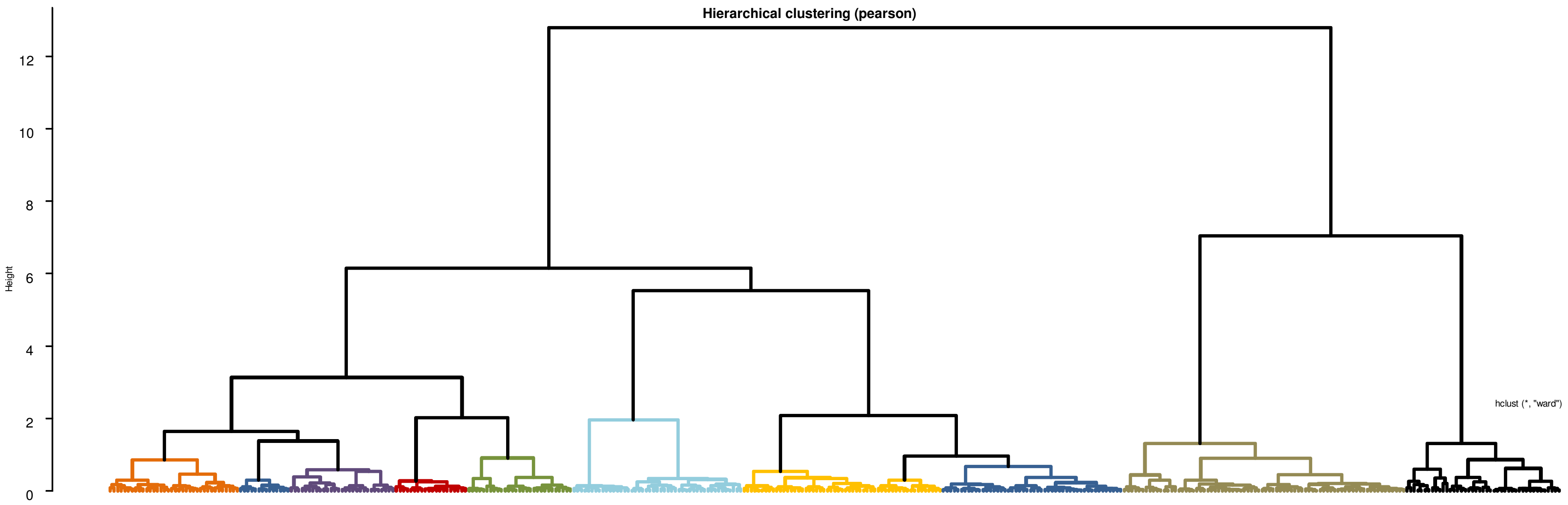

Study nr	1	2	3	4	5	6	7	2	8	2 & 6
Sample location	Large intestine	Small intestine	Large intestine	Large intestine	Large intestine	Large intestine	Large intestine	Large intestine	Large intestine	Small intestine and large intestine of 2 week old mice
Mouse strain	C57BL/6J and C57BL/6J-KO	C57BL/6J	C57BL/6J and BalB/c	C57BL/6J	C57BL/6J and 129Sv	$Muc2^{-/-}$, $Muc2^{+/-}$ and WT 129Sv	C57BL/6J	C57BL/6J	C57BL/6J	C57BL/6J and $Muc2^{-/-}$, $Muc2^{+/-}$ and WT 129Sv
Facility location	Wageningen University	Wageningen University	University Medical centre Groningen	University Medical centre Groningen	University of Copenhagen	Wageningen University	Wageningen University	Wageningen University	University of Copenhagen	Wageningen University
Supplier	Erasmus University Medical Centre, the Netherlands	Charles River, L'Arbresle, France	Harlan, Horst, the Netherlands	Charles River, L'Arbresle, France	Taconic Europe	Erasmus University Medical Centre, the Netherlands	Charles River Laboratories, Maastricht, the Netherlands	Charles River, L'Arbresle, France	Taconic Europe	Charles River, L'Arbresle, France
Gender	Female	Male	Male and female	Male	Male	Male and female	Male	Male	Female	Male and female
