# Supplementary figures and images for: Mouse models for human intestinal microbiota research: a critical evaluation
Source: Cell Mol Life Sci. 2017 Nov 9;75(1):149–60. doi: 10.1007/s00018-017-2693-8 (PMC5752736; doi:10.1007/s00018-017-2693-8)

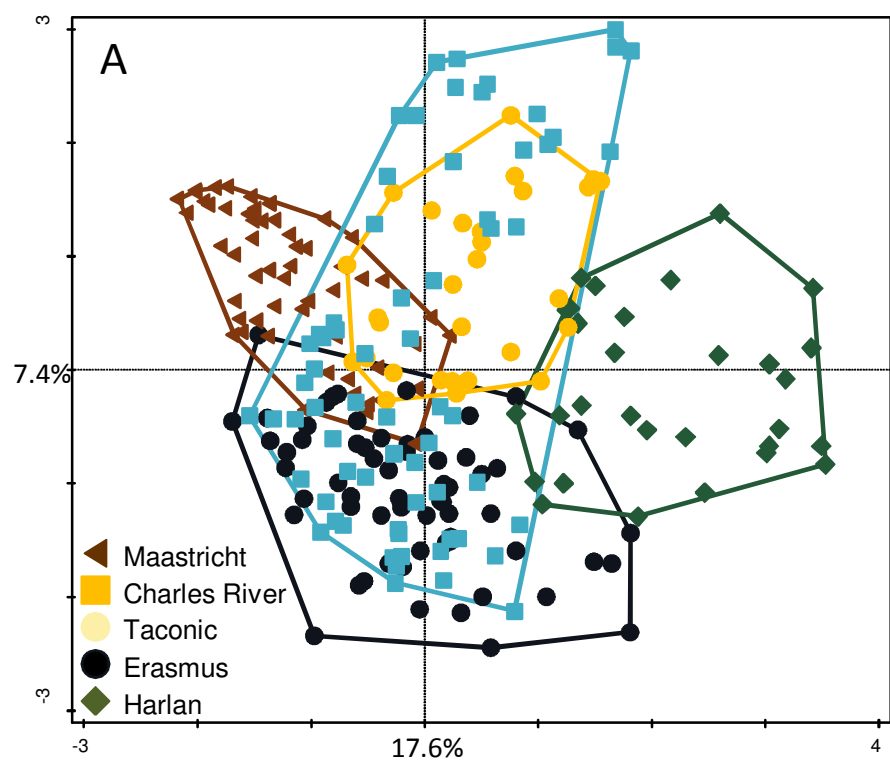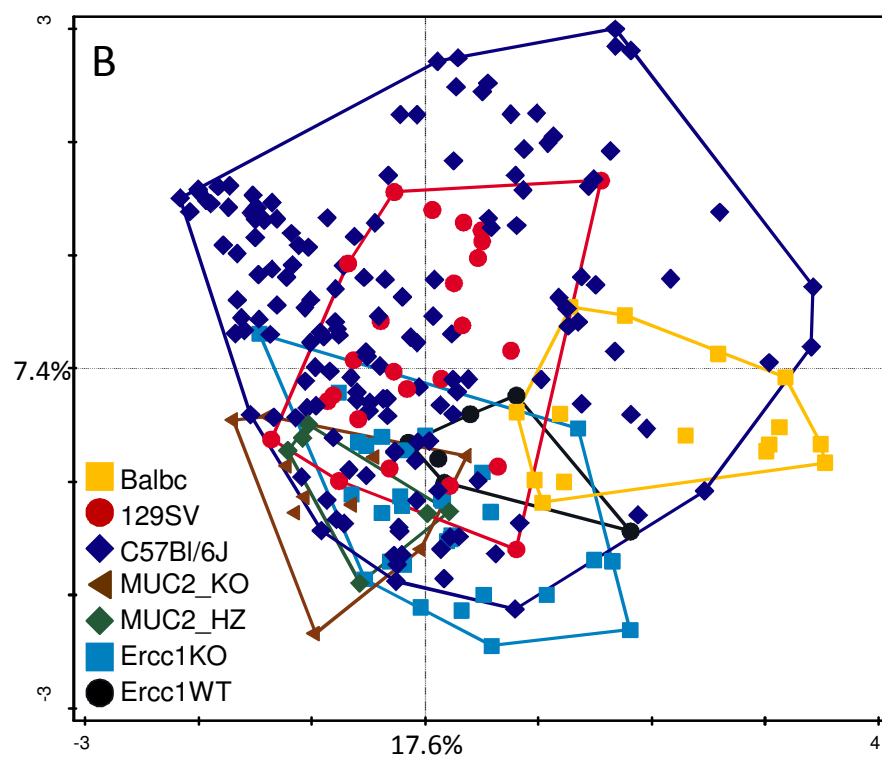

Supplement: Supplementary file 2 — Supplementary Figure S2. Redundancy analysis of the large intestine samples of seven studies, containing a total of 244 samples. Genotype, facility and provider are taken along as variables for the analysis and explain 43.5% of the data. Here the clustering is shown of the different providers (A) and of the strains (B). In Table 1 are the significant variables shown (PDF 127 kb) [file 18_2017_2693_MOESM2_ESM.pdf]
